# Preparing physiotherapists for the future: the development and evaluation of an innovative curriculum

**DOI:** 10.1186/s12909-024-06537-1

**Published:** 2025-01-17

**Authors:** Niki Stolwijk, Anne van Bergen, Evy Jetten, Marjo Maas

**Affiliations:** 1 HAN University of Applied Sciences, Academy Allied Health Sciences, Nijmegen, The Netherlands; 2 Radboud University Medical Centre, Scientific Institute for Quality of Healthcare (IQ Health), Nijmegen, The Netherlands; 3 FysioSupport, Mill, The Netherlands; 4 Department of Orthopedic Surgery, VieCuri Medical Centre, Venray, The Netherlands

**Keywords:** Health professional education, Innovation, Self-directed learning, Physiotherapy, Communities of practice

## Abstract

**Background:**

Educational innovation in health professional education is needed to keep up with rapidly changing healthcare systems and societal needs. This study evaluates the implementation of PACE, an innovative curriculum designed by the physiotherapy department of the HAN University of Applied Sciences in The Netherlands. The PACE concept features an integrated approach to learning and assessment based on pre-set learning outcomes, personalized learning goals, flexible learning routes, and programmatic assessment. PACE distinguishes itself from traditional education because of the flexible learning routes, vertical organization in learning communities, absence of pre-defined learning activities and class schedules, and a culture of continuous learning and development. PACE is based on three guiding principles: 1) flexible and varied, 2) self-directed and collaborative, 3) future-oriented. PACE was implemented in 2021 for first-year students. This study evaluates the implementation to inform future curriculum development.

**Methods:**

A sequential explanatory mixed methods design was used to evaluate the implementation of PACE using a questionnaire, focus groups, in-depth interviews, and a national progress test allowing for benchmarking results. Participants were undergraduate physiotherapy students of cohort 2021–2022, the first group who experienced PACE and teachers involved with this cohort. Questionnaire data were analyzed using descriptive statistics. To compare mean total scores of the national progress test between four different universities a one-way ANOVA was conducted including a post-hoc analysis. Reflexive thematic analysis guidelines were applied to analyze the interview data.

**Results:**

In total 82 first year students (44,6%) of cohort 2021–2022 and 36 teachers (60%) completed the questionnaire. Results show that the guiding principles were implemented as intended. Results of the national progress test on knowledge and clinical reasoning showed that students of the HAN University performed well compared to other universities. Thematic analysis of interviews and focus groups resulted in three themes and nine subthemes: 1) navigating a personalized curriculum, 2) caring and sharing, and 3) shaping professional identity. PACE contributed positively to students' intrinsic motivation, learning joy, identity development, and life-long learning skills. Areas for improvement were self-directed learning support, and teaching strategies to prompt deep learning.

**Conclusion:**

The evaluation showed that the guiding principles of PACE were implemented as intended and that the innovation positively contributed to student learning,

**Supplementary Information:**

The online version contains supplementary material available at 10.1186/s12909-024-06537-1.

## Background

Health professional education is a dynamic field that continually evolves to meet the challenges of rapidly changing healthcare systems and societal needs. In the Netherlands, as in many countries, the sustainability of healthcare is under pressure due to an aging population, increasing complexity of health conditions, and a shortage of healthcare workers [1]. To maintain the quality, accessibility, and affordability of healthcare, healthcare workers need to develop new competencies and adopt new roles with an emphasis on continuing professional development, communication, and interprofessional collaboration skills [2].

The physiotherapy department of the HAN university of Applied Sciences in the Netherlands actively embraced this challenge through innovative educational practices. A design-based research approach was adopted to develop a new curriculum [3, 4]. This involved a comprehensive process including focus groups, interviews, and design sessions with all relevant stakeholders to develop a shared vision on physiotherapy and physiotherapy education. This vision was combined with the best evidence on education and assessment, and new insights gained from the successful implementation of the Delta-stream, an experimental curriculum variant [5–7]. These efforts led to the development of PACE (Physiotherapy Advanced Collaborative Education) program.

The PACE concept features an integrated approach to learning and assessment based on pre-set learning outcomes, personalized learning goals, flexible learning routes, collaborative learning, self-directed learning support, involving continuous, multi-source feedback according to the principles of programmatic assessment [8, 9]. Learning outcomes are clustered in units of learning outcomes and linked to professional roles (Table 1 presents an example). The PACE concept distinguishes itself from traditional education through its vertical organization in communities of practice (CoP) comprising all academic years, and the absence of pre-defined learning activities and class schedules. Learning within a community of practice focuses on sharing knowledge, professional norms and values, and collaboratively working on solutions to complex real-life problems from different perspectives and levels of expertise according to the concept of Gruess et al. [10]. See Table 2 for more details.

**Table 1:** Example of two learning outcomes linked to the professional role ‘care provider’

Professional role	Unit of Learning Outcomes	Learning Outcomes
**The care provider**	**Diagnosis**	1.1* Screening* The student determines, within a limited time, whether there is a pattern of signs and/or symptoms within the competence of an individual physical therapist by asking targeted questions, conducting tests, or performing other diagnostic procedures. The student screens for both general and patient-group-specific red flags, thereby ruling out serious injuries. The student appropriately informs the client about the findings of the screening. 1.2 *Anamnesis* The student systematically investigates the health issue, applying relevant knowledge and health models. The student demonstrates a professional attitude, uses appropriate communication skills, and employs suitable measurement instruments to clarify the client’s request for help and assess the movement-related problem. Based on the collected data, the student organizes and classifies the information, methodically, formulates appropriate hypotheses, and develops a physical examination plan. 1.3 *Physical Examination* etc. 1.4 *Physiotherapeutic Diagnosis* etc.
	**Treatment**	
**The health promotor**	**Prevention**	
**The reflective professional**	**Life-long learning**	
**The innovative professional**	**Applied research and innovation**

**Table 2:** Guiding principles of the PACE concept

Guiding Principle	Description	Operationalization
**Flexible & varied**	Pre-defined learning outcomes including performance criteria assessed according to the concept of programmatic assessment [9, 11]. Personalized learning goals and flexible learning paths tailored to the ambitions and capabilities of students.	Teachers are adaptive to student’s learning needs and preferences. Except for a few large group lectures and planned standardized assessment (knowledge tests), there are no class schedules. The digital learning environment is designed according to the principles of blended learning including video-cases, lectures, e-learnings, e-books, e-journals, simulations, demonstrations, and practice assignments.
**Self-directed & collaborative**	Self-directed learning (SDL) involves self-regulation and metacognition [12]. Students self-direct their learning process by setting goals, choosing learning activities, self-monitoring study progress and continuously asking for feedback from peers, senior students, clinical supervisors and teachers [11, 13].	Teachers have a coaching role in supporting SDL and collaboration among all members of the CoP. They collectively monitor the study progress of their students on a regular basis. The student is in the lead for asking for feedback on written products of performances, and choosing a feedback provider. Feedback is sampled via electronic feedback forms for different learning outcomes, including performance criteria that can be scored on a 5-point Likert scale underpinned by narrative feedback.
	Learning in a CoP, comprising students from all academic years, field supervisors and a fixed group of teachers. Collaboratively working on authentic problems to promote a deep understanding through constructive dialogue.	Bachelor students (*n* = 750) are assigned to 6 CoP’s. Each CoP is supervised by 8–10 teachers with various expertise (e.g. neurology, orthopedics, geriatrics).
	Demonstrating learning outcomes in a portfolio.	Assessment scores and narrative feedback of all feedback providers are collected in an electronic portfolio, aggregated, and graphically displayed in the portfolio dashboard, allowing for easy self-monitoring study progress on different learning outcomes.
**Future-oriented**	A curriculum that optimally prepares students for a changing healthcare system and evolving competencies and roles. Working on open-ended, authentic, complex problems stimulating students to deal with uncertainty regarding the outcomes	Teachers approach students as future colleagues and set ambitious standards [6, 14]. Problems are directly derived from clinical practice in the form of video-recordings of patients or professionals with an emphasis on themes as chronic diseases, cost-effective and client-centered interventions, prevention of health disparities, technology, and interprofessional collaboration [15]. Culture of continuous learning and development on the level of the individual, team, and organization [16].

PACE is built on three guiding principles: 1) flexible & varied, 2) self-directed & collaborative, and 3) future-oriented.

The guiding principles are operationalized in the curriculum and the organization as shown in Table 2. The development of the new curriculum was accompanied by an extensive faculty development program to prepare teachers and staff for their new competencies and roles regarding didactics and coaching skills, programmatic assessment, and team development [16]. In July 2024, the physiotherapy department of the HAN university received the Dutch NRO reward for Educational Innovation in Higher Education (https://www.onderwijskennis.nl/kennisbank/pace-samen-leren-samen-werken).

The educational innovation PACE was implemented in 2021, for first-year students and in 2022 for second-year students. The aim of this study was to evaluate the implementation of PACE and to inform future curriculum development. Our research questions were:

- Is PACE implemented as planned and what is needed to support further implementation?
- What are the learning results of HAN students compared to students of other universities?
- How is PACE perceived by students and teachers?

## Methods

### Design

A sequential explanatory mixed methods research design was used to evaluate the implementation of PACE using questionnaires, focus groups, in-depth interviews, and a national progress test on knowledge and clinical reasoning. Focus groups and interviews were used to elaborate on the questionnaire results. The data collection period was between June 2022 and December 2022. The checklist Consolidated criteria for Reporting Qualitative research (COREQ) was used to design and report the study [17].

### Participants

Physiotherapy students of cohort 2021–2022, the first group who experienced PACE and teachers involved with this cohort.

### Data sampling

#### Questionnaires

A 22-item questionnaire was developed by our research team (MM, EJ, AB, and NS) for students and teachers, based on the three guiding principles of PACE: 1) flexible & varied (7 items), 2) self-directed & collaborative (10 items), 3) future oriented (5 items). These items operationalized the guiding principles across four domains: curriculum content, assessment, study coaching, and facilities. The questionnaire used a five-point Likert scale ranging from “strongly disagree” to “strongly agree,” including a “not applicable” option and an open field for comments. See Supplementary file 3.

All first-year students of cohort 2021–2022 and teachers were invited via Microsoft Teams (https://teams.microsoft.com/v2/), the student communication platform. The questionnaire was administered via Microsoft Forms (https://www.microsoft.com/en-us/microsoft-365/online-surveys-polls-quizzes). A digital link to the questionnaire was sent in June 2022. All participants who were willing to participate, gave their online informed consent before completing the questionnaire.

#### National progress test

To get insight into students’ performance and to evaluate the educational innovation we looked at the scores of the National Progress Test Physiotherapy (NPTP). The NPTP is an innovative, standardized online assessment, administered two times a year to physiotherapy students of all undergraduate levels from four universities in the Netherlands. It covers a wide range of knowledge and clinical reasoning skills across various domains such as anatomy, physiology and a variety of conditions. The longitudinal and repetitive nature of the NPTP should encourage knowledge retention, discourage last minute superficial learning [18, 19]. The NPTP is designed to provide students feedback on their study progress compared to their peers and enables to evaluate education programs nationwide by comparing scores across different cohorts and institutions [20]. The Progress Test is a formative test, embedded in the assessment program [18], and the results are imported in student portfolios. The NPTP contains 100 multiple choice items, related to approximately 35 clinical cases. Test scores can vary from 0 to 100.

#### In-depth interviews and focus groups

In-depth interviews with students and focus groups with teachers were conducted in Fall 2022. When students entered their second year, they reflected on their experiences with PACE in their first year. We used purposive sampling, aiming for maximal variation in terms of gender, and CoP. Data collection continued until saturation was reached. We used in-depth interviews to ensure safety and enhance reflection. A semi-structured interview guide was developed by MM and NS for students and teachers (Supplementary file 1 and 2). They received an information letter detailing the interview's purpose and procedure and signed an informed consent. In-depth interviews with students were conducted by AvB face-to-face or online. Focus groups with teachers were conducted by AvB and EJ. AvB facilitated the focus group discussions, EJ took notes. The in-depth interviews and focus groups were audio recorded and transcribed verbatim. Transcripts of the interviews were returned to the students and teachers for a member check to enhance the credibility of the findings [17, 21, 22].

### Data analysis

#### Questionnaires

Questionnaire data were exported from Microsoft Forms, imported into IBM SPSS statistics 25, and then anonymized accordingly. Median scores, including interquartile ranges (IQR), were calculated and described for each questionnaire item. Mean scores and standard deviations (SD) were calculated and described for each guiding principle (cluster of items), as item aggregation within each domain increased measurement reliability and resulted in approximately normal distributions, supporting the use of means and standard deviations. Differences between students and teachers regarding the guiding principles were analyzed using independent samples t-tests, with corresponding *p*-values reported.

#### National progress test

Test scores of the NPTP cohort 2021–2022 administered in May 2022, were entered in IBM SPSS 25. Mean sum scores and standard deviations are calculated and described of undergraduate first year students of all universities. To compare mean total scores between four different universities a one-way ANOVA was conducted including a post-hoc analysis. Post hoc tests allow for testing difference between multiple group means while also controlling for type-1 error rate [23].

#### Interviews and focus groups

All transcripts were entered in Atlas-ti Software version 23. The data were analyzed according to reflexive thematic analyses guidelines described by Braun and Clarke [21, 22]. The guiding principles of PACE were used as pre-defined codes according to our research question. We took an ‘inductive’ approach to analyze the data and to identify emerging themes (patterns of shared meaning), and subthemes. First, a set of transcripts of student interviews and focus groups were studied by AvB, MM, and NS individually and relevant text fragments regarding the research questions were coded. Accordingly, codes were discussed, and a code book was developed based on consensus to support the analysis of all the transcripts. By discussing and comparing codes during several consensus meetings, categories of codes were developed, and themes were identified that emerged from the data. We consciously and explicitly reflected on our personal viewpoints and theoretical lenses and discussed differences to ensure reflexivity [21].

## Results

### Participants

In total 82 first year students (44,6%) of cohort 2021–2022 and 36 teachers (60%) from six different CoP’s completed the questionnaire. Table 3 shows participant features. Interviews were conducted with 12 students, two from each CoP. The focus groups consisted of 12 teachers.

**Table 3:** Participants characteristics

	**Students (*n* = 82)**	**Teachers (*n* = 36)**
**Gender, female; n (%)**	50 (61)	19 (52,8)
**Age in years; median (Q1, Q3)**	19 (18–21)	36,5 (28–44,75)
**Working experience as a physiotherapy teacher (years)**		
0–5	0	20
6–10	0	5
11–15	0	7
> 15	0	4

### Questionnaire

Looking at the summary of the questionnaire results (Table 4), students and teachers agree on the implementation of the guiding principles. Overall, students and teachers score high (median of 4 or 5 on a scale of 1 to 5) on the underlying items per guiding principle. Table 5 shows the complete questionnaire. In four of the 22 items the median score was 3. Regarding the first principle, flexible and varied, students were significantly less convinced than teachers.

**Table 4:** Mean scores subdomains questionnaire

Guiding principles	Students *N* = 82		Teachers *N* = 36		*P*-value
	**mean ± SD**	**min – max**	**mean ± SD**	**min – max**	
**Flexible & varied**	3,68 ± 0,42	2,86 – 4,86	3,96 ± 0,39	3,00 – 4,43	< 0,001
**Self-directed & collaborative**	3,80 ± 0,41	2,80 – 4,90	3,87 ± 0,33	3,10 – 4,50	0,192
**Future-oriented**	3,68 ± 0,47	2,80 – 5,00	3,79 ± 0,52	2,80 – 5,00	0,245

**Table 5:** Questionnaire results: student and teacher perspectives. Items are presented from the student perspective, with teachers answering equivalent questions from their viewpoint. Responses, scored on a 1–5 Likert scale (1 = strongly disagree, 5 = strongly agree), are grouped by guiding principles, showing median scores and interquartile ranges

Guiding Principle Questions	Students		Teachers	
	Median	IQR	Median	IQR
**Varied & flexible**				
1. In education we work with varied clinical cases as they occur in professional practice.	4,00	0 (4–4)	4,00	0 (4–4)
2. I can choose from a variety of clinical cases or learning activities and thus determine my own learning route.	4,00	1 (3–4)	4,00	1 (3–4)
3. I can demonstrate the learning outcomes in my portfolio in various ways (product/video/performance etc.).	4,00	1 (4–5)	5,00	1 (4–5)
4. I can decide when I ask for feedback and in what form (written/conversation/combination).	4,00	1 (3–4)	4,00	1 (4–5)
5. I can decide when my portfolio is ready for assessment.	4,00	1 (3–4)	4,00	1 (3–4)
6. I experience sufficient variation in the learning activities such as lectures, workshops, written assignments, patient demonstrations.	3,00	2 (2–4)	4,00	1 (3–4)
7. Learning coaches respond to my personal learning questions and meet my learning needs.	4,00	1 (3–4)	4,00	1 (4–5)
**Self-directed & collaborative**				
8. I am encouraged to take responsibility for my own learning process.	4,00	1 (4–5)	5,00	1 (4–5)
9. I experience the added value of learning with students from different years (vertical CoP's).	4,00	1 (3–4)	4,00	1 (4–5)
10. I receive useful feedback from teachers to improve my performance.	4,00	0 (4–4)	4,00	0 (4–4)
11. I receive useful feedback from senior students to improve my performance.	4,00	0 (4–4)	4,00	1 (3–4)
12. I receive useful feedback from peers (same study year) to improve my performance.	4,00	1 (3–4)	4,00	1 (3–4)
13. I critically reflect on the feedback I have received and show what I have done with it.	4,00	0 (4–4)	3,00	1,50 (2,25–3,75)
14. I feel safe in my learning group to share my learning experiences and demonstrate my learning results.	4,00	1 (4–5)	4,00	1 (4–5)
15. My teachers have sufficient insight into my learning process.	3,00	1 (3–4)	4,00	1 (3–4)
16. I feel connected to the students and teachers of my Community of Practice (CoP)	4,00	1 (3–4)	4,00	1 (4–5)
17. I actively contribute to learning within my COP.	4,00	1 (3–4)	4,00	1 (3–4)
**Future-oriented**				
18. I get a clear picture of the future physiotherapist	4.00	1 (3–4)	4,00	1 (3–4)
19. I am being prepared to adapt to the changing roles and tasks of the future physiotherapist.	4,00	1 (3–4)	4,00	1 (3–4)
20. I develop learning skills for life-long learning.	4,00	0 (4–4)	4,00	0,75 (4,00–4,75)
21. I am introduced to new technology for education and healthcare.	3,00	1 (3–4)	3,00	1 (3–4)
22. Teachers approach me as a future colleague physiotherapist.	4.00	1 (3–4)	5.00	1 (4–5)

### National progress test

Table 6 shows that mean test scores of the HAN university students are satisfactory compared to other universities. The one-way ANOVA showed a statistically significant difference between groups, F(3,786) = 88.06, *p* < 0.001. A Tukey post hoc test revealed that the mean total scores of HAN university students (51,30 ± 8,48) was significantly higher than mean total scores of students from university three and four (37,66 ± 10,69, *p* < 0,001 and 39,26 ± 12,05, *p* < 0,001 respectively) (see Table 7). Chrohbach’s alpha for internal consistency was 0,81.

**Table 6:** Total scores of national progress test

May-22		
Propedeuse	**N**	**Mean total score ± SD**
HAN	163	51,30 ± 8,48
University 2	200	49,53 ± 10,25
University 3	261	37,66 ± 10,69
University 4	166	39,26 ± 12,05

**Table 7:** Results of post-hoc Tukey test

ANOVA Propedeuse			95% Confidence Interval			
	**University**	**Mean Difference**	**Std. Error**	**Sig**	**Lower Bound**	**Upper Bound**
**HAN**	2	1.78	1.11	0.376	-1.07	4.62
	3	13.64^a^	1.05	< ,001	10.95	16.33
	4	12.04^a^	1.16	< ,001	9.07	15.02

^a^The mean difference is significant

### In-depth interviews and focus groups

Thematic analysis of interviews and focus groups resulted in three themes and nine subthemes as presented in Table 8. Each theme and subtheme is described and illustrated with quotes from students and/or teachers.

**Table 8:** Themes and subthemes emerged from the interview data (students and teachers)

Themes	Subthemes
**1. Navigating a personalized curriculum**	Embracing ownership
	Adapting teaching practices
	Bridging the culture gap
**2. Caring and sharing**	Fostering peer learning
	Building confidence
	Sustaining engagement
**3. Shaping professional identity**	Life-long learning
	Embracing spontanious learning
	Envisioning the future

### Theme 1. Navigating personalized learning

#### Embracing ownership

The flexible program structure allowed students to personalize their learning paths, choosing which classes and activities to attend and which ones to skip for the benefit of other learning activities that better match their learning needs and preferences. This autonomy fosters a sense of ownership over the learning process.

*S3: “That you can choose, like this class isn’t very relevant to me, I already know a lot about it, so I choose to do something else […], that works well for me.”*

*T11: “I notice that students are becoming more comfortable with planning what, when, and where they want to learn, and what they need from me, today or tomorrow, and they can easily switch between them.”*

However, some students question if the curriculum contributes to deep learning and critical thinking skills. Students and teachers mentioned the risk of a superficial approach to learning.

*S5* *:* *“Yes, it [SDL] has its advantages and disadvantages, I think when it's so varied, you can learn a bit of everything, but if there’s too much, you might lose the opportunity to really dive deep into a particular subject. Of course, I can steer my own learning, but I also want to study all the cases.”*

*S10: “Because you have so much freedom of choice, it can sometimes be difficult to truly immerse yourself in the material. You might feel like, well, I'll just learn the basics, and that’s enough,’ and then you move on.”*

#### Adapting teaching practices

While the flexible curriculum encourages innovation, some teachers found it challenging to depart from traditional pre-structured curriculum content and pre-defined teaching methods, often reverting to familiar approaches. To align with student’s learning needs and preferences, flexibility and creativity is needed. Teachers highlighted the need for ongoing professional development and critical reflection on current teaching practices.

*T2: “In terms of didactic methods, I try to vary, although I don’t always succeed in doing so. I tend to choose methods that feel most comfortable for me. I really must consciously remember to vary, there is room for improvement for me.”*

*T10* ***:*** *“I believe that we are all still learning to vary our approaches and to make learning more challenging and stimulating. Besides offering different content or contexts as we have done, we need to think about how we can shape it didactically in different ways.”*

#### Bridging the culture gap

The program aims to develop students' SDL skills, enabling them to set personal goals, create learning strategies, and reflect on their progress, ask for feedback and decide on the next step. Successful students transition from extrinsically motivated tasks to intrinsically driven learning, e.g. from completing pre-defined assignments and asking for general feedback to completing self-chosen assignments and asking for personalized feedback. For many students, particularly those transitioning from a more structured high school environment, the shift to a self-directed learning approach was challenging. They reported feeling overwhelmed and needed additional support to navigate the curriculum.

*S5: “In the beginning, I needed to get used to it. The freedom and flexibility felt like a culture shock. I really had to get used to it.”*

*S6: “At first, I didn’t know what to do, and there was no guidance. But now that we understand it better, it’s motivating. It really encourages you to work from your own motivation.”*

*S4: “Last year, I was focused on completing video cases [problem tasks], just to fulfill requirements. This year, I’m more centered around identifying my own needs and desires in the process.”*

### Theme 2: Caring and sharing

#### Fostering peer learning

Second-year students expressed a sense of responsibility and enjoyment in helping first-year students. This peer tutoring not only aids the juniors but also reinforces the tutors’ own understanding and skills where junior students profited from role modeling opportunities.

*S3: “It’s rewarding to help first-year students in their process, while simultaneously strengthening my own knowledge.”*

*S5: “I find it fantastic to help first-year students. Collaborating across different academic years is great because you can always learn from each other.”*

#### Building confidence

Teachers observed that students’ confidence and self-efficacy beliefs grew significantly through vertical collaboration, where senior students mentor junior students. This process helps students progress through Miller’s pyramid of clinical competence, moving from ‘knowing’ to ‘showing how.’

*T4: “The growth in self-confidence is evident. Students who struggled in their first year are now starting to shine, showcasing their increased knowledge and ability to articulate concepts.”*

*S2: “Assisting first-year students provides reassurance, affirming that I already know quite a lot. With third years, it's like a benchmark; am I on the right track?”*

#### Sustaining engagement

While collaboration within the Community of Practice (CoP) is beneficial, maintaining engagement among senior students is challenging due to their reduced presence on campus for internships or minors [out of campus programs]. This lack of interaction limits the exchange of experiences across different academic years.

*T12: “Collaboration among first- and second-year students is smooth, but engaging senior students is challenging. Although they initially participate during onboarding [introduction of a learning period], sustaining this involvement is difficult as time progresses.”*

*S10: “Obtaining [online] feedback from senior year students [on written products or video-recorded performances] is no problem; however, their physical presence is limited.”*

### Theme 3: Shaping professional identity

#### Life-long learning

Students’ and teachers’ experiences show that PACE contributes to the development of life-long learning skills such as, dealing with uncertainty and navigating complex real-world situations, that future physiotherapist need to respond to the increasing complexity of help requests in clinical practice.

*S12: “It is beneficial that I learned how to plan and structure my own learning path. These are skills that I need as a physiotherapist but also in daily life. I am confident to use these skills in the future.”*

*T7: “I think the current education is future-oriented because students learn to handle different things like chaos, complexity, making their own choices, which is very important as a physiotherapist. Students are being prepared for the ‘real world’.”*

#### Embracing spontaneous learning

What both teachers and students see as an advantage of a flexible program is that the curriculum is not overloaded with prescheduled classes. Instead, there is room for alternative, spontaneous learning activities organized by students. By bringing real-life experiences into the classroom, students took the lead in bridging the gap between theory and practice, strengthening their engagement with the learning content.

*S2: “[...] Last week, a girl from my handball team got injured, and I took her to school the next day. Her problem didn’t fit into the program, but all the teachers were super enthusiastic, and that’s when you learn the most.”*

*T2: “As a teacher I also feel the flexibility in education. Students were working on a clinical case on a hip disorder, when a student said; “my grandmother has hip osteoarthritis and a lot of difficult questions [that I couldn’t answer].” So, the next week we had a grandmother in the classroom.”*

#### Envisioning the future

The program’s flexibility allows students to explore various physiotherapy specializations and work settings. From the first year, students are encouraged to envision their future as a physiotherapist, to anticipate their future role in society in order to build a realistic image of the profession and a personal, professional identity.

*S4: “Teachers share their experiences from different physiotherapy settings. They are interested in what I want to do after graduation and offer guidance on finding the most suitable career path.”*

*T8: “We encourage students to take their own path and help them become the physiotherapist they want to be. The focus is more on discovering what kind of physiotherapist they want to become and how they will achieve that.”*

## Summary of the results

In Table 9 the strengths of the PACE concept in the perspectives of students and teachers, including the areas that need improvement are summarized.

**Table 9:** Strengths and areas for improvement of PACE

Theme		Strength	Areas for improvement
**1**	Navigating the curriculum	Opportunity to personalize the learning path fosters a sense of ownership over the learning process, increasing intrinsic motivation te learn.	Need for tailored coaching to support the development of self-directed learning skills and bridge the gap between teacher-oriented learning and self-directed learning.
			Need for ongoing professional development regarding new teaching approaches to align with student needs and preferences.
			To reduce the risk of a superficial approach to learning.
**2**	Caring and sharing	Vertical collaboration and peer tutoring contributes to learning joy, shared responsibility, and increased self-efficacy beliefs.	Need for sustained engagement of senior students to foster collaboration across different academic years.
**3**	Shaping professional identity	PACE contributes to the development and life-long learning skills such as dealing with uncertainty and navigating complex real-world situations.	Need for the development of critical thinking skills to enhance deep learning.
		Making room for real-life experiences in the classroom, stimulates the transfer of learning and engagement with the learning content.	
		Encouraging students to envision their future contributes to professional identity building.	

## Discussion

This study aimed to evaluate the implementation of PACE educational innovation, which was introduced for first-years physiotherapy students in 2021. Our goal was to assess whether PACE was implemented as planned, compare learning outcomes of HAN students to those of other universities and understand how students and teachers perceive PACE. The innovation was evaluated with questionnaires, a national progress test allowing for benchmarking results, focus groups, and in-depth interviews. The insights gained from this evaluation are intended to guide future curriculum development.

The findings of the questionnaire showed that PACE was largely implemented as intended, with both teachers and students acknowledging the integration of the three guiding principles in the curriculum: 1) flexible & varied, 2) collaborative & self-directed and 3) future-oriented. However, students were less convinced than teachers on the successful implementation of the first principle. This difference in perception could be attributed to some teachers struggling to fully adapt to the innovative method, adequately respond to student needs and tending to fall back into traditional teaching approaches. The challenge to adequately prepare teachers for an educational innovation and to keep them on track is well described in the literature on innovation in higher education [24].

The results from the National Progress Test show that HAN students performed comparably well in knowledge acquisition and clinical reasoning compared to their peers at other physiotherapy universities. Apparently, students are capable of developing relevant knowledge and transferring this knowledge to solve clinical cases without knowing the assessment content in advance. We hypothesize that the PACE concept better aligns with learning preferences and abilities of Generation Z, defined as individuals born between 1990 and 2010s. The literature shows that todays’ students are not interested in simply showing up for class, sitting through a lecture, and taking notes that they’ll memorize for an exam later on. Instead, they expect to be fully engaged, prefer collaborative learning, not limited to in-person interactions. Generation Z is completely comfortable with learning alongside other students, even outside of their own school. Moreover, they have an affinity for seeking information through video [25]. The PACE concept aligns with these preferences, offering students ‘choice and voice’.

We identified three major themes from the interview data and nine subthemes and summarized the results by describing the strengths of PACE and the areas for improvement.

Regarding the first theme ‘*navigating the curriculum’* referring to the concept of SDL, PACE contributed to feelings of ownership over the learning process and intrinsic motivation to learn according to the perceptions of students and teachers. However, the data also showed that students in their first year needed more coaching and support to develop necessary SDL skills featuring the PACE concept such as (setting goals, planning learning activities, monitoring and evaluating results), to bridge the gap between teacher-oriented and learner-oriented approaches. Taking responsibility for ones learning is not self-evident [12]. SDL skills are metacognitive skills that are related to self-regulation skills. Jolles [26] emphasizes that the development of self-regulation skills is closely linked to brain development, particularly in the prefrontal cortex, which is responsible for executive functions such as planning, decision-making, and impulse control These skills and the corresponding brain regions continue to mature well into adolescence and early adulthood. Experiences and learning opportunities play a significant role in shaping these skills. When students have not yet mastered these skills, SDL is hindered. Two useful strategies to guide students to SDL are ‘modeling’ and ‘scaffolding’. With modeling, teachers (or peers) show students how they use their metacognitive skills when completing an assignment or task [27, 28]. Scaffolding refers to the strategy by which teachers provide students with cognitive supports early in their learning, and then gradually remove these supports as students develop more knowledge and skills. Scaffolding requires customization and support on an individual level. Given the context of undergraduate physiotherapy education (the youngest students are 17–18 years old), ongoing professional development of teachers is needed to support SDL.

Looking at the second theme ‘*caring and Sharing’* referring to collaborative learning, PACE contributed to learning joy and increased self-efficacy beliefs. These findings align with the self-determination theory of Ryan & Deci on intrinsic motivation [29]. They explain that intrinsic motivation depends on three psychological needs: autonomy, competence and relatedness. The PACE concept allows students to draft a personal learning path (autonomy), collaborate intensely with peers and experts within their CoP (relatedness) and develop self-efficacy beliefs (competency) by peer tutoring and peer assessment. The findings also align with a study on peer assessment in physiotherapy education, showing the importance of observation and role-modeling peers for skills development [30]. Although, the data show that peer tutoring strengthens learning, sustained engagement of senior students was challenging. It is understandable that senior students balanced their commitment with peer learning and the efforts needed (time and costs for travelling). Cook and Artino [31] added to the self-determination theory the psychological need ‘perceived value’ as an important motivator. Commitment and shared responsibility are important conditions for collaborative learning within a CoP [28, 32, 33]. Creating added value for senior students is one of the challenges for the improvement of the PACE concept.

Regarding the third theme, s*haping professional identity*, PACE contributes to the development of life learning skills such as critical thinking, dealing with uncertainty and navigating complex real-world situations. This is an important finding regarding the fact that approximately 45 percent of graduated physiotherapists leave the profession within five years. Reasons are high workload, uncertainty of the quality of care provided, low feelings of self-efficacy and financial uncertainty [34]. Cruess et al. [35] described factors that contribute to professional identity formation such as: providing a welcoming community, assisting students in their process of identity development, role modeling and experiential learning. This is in line with the PACE concept. In PACE teachers enhance collaboration within the CoP, allow for real-life experiences in the classroom to stimulate the transfer from theory to practice, and encourage students to build a realistic image of their profession. Trede & McEwen (2012) argue that professional identity formation is intertwined with the process of ‘professional enculturation’, as students adopt the values, language, and behaviors that characterize their future profession [14, 36]. We hypothesize that students who completed the PACE program will demonstrate greater professional enculturation and be better equipped to position themselves as professionals in the healthcare landscape. Future research needs to demonstrate if this hypothesis is correct.

## Conclusion

This study evaluated the implementation of the educational innovation PACE and identified its strengths and weaknesses. The evaluation showed that the guiding principles of PACE were implemented as intended. PACE contributed positively to students’ intrinsic motivation, learning joy, identity development, and life-long learning skills. However, there is a need for additional SDL guidance, particularly for entering first-year students, and strategies to prompt deep learning. Future research should explore strategies to enhance teacher adaptation to innovative teaching methods and ensure sustained engagement across all student levels. With this educational innovation, we took a risky but well-founded step towards future-proof education and healthcare.

## Supplementary Information

Supplementary Material 1.

Supplementary Material 2.

Supplementary Material 3.

## Data Availability

All anonymized data are available on request. No datasets were generated or analysed during the current study.

## Abbreviations

CoP: Community of practice
SDL: Self-directed learning
OLE: Online learning environment
IQR: Interquartile range
NPTP: National progress test physiotherapy
PACE: Physiotherapy advanced collaborative education
SD: Standard deviation
