# Supplementary material for: Preparing physiotherapists for the future: the development and evaluation of an innovative curriculum
Source: BMC Med Educ. 2025 Jan 17;25:83. doi: 10.1186/s12909-024-06537-1 (PMC11740659; doi:10.1186/s12909-024-06537-1)
Supplement: Supplementary file 1 — Supplementary Material 1. [file 12909_2024_6537_MOESM1_ESM.docx]

**Additional file 1: Interview guide students**

**Flexible**

- How do you experience flexibility in education?
- Do you have a concrete example of the flexibility in education?
- What is the impact of flexibility on learning?

**Varied**

- How do you experience variety in education?
- Do you have a concrete example of variety in education?
- What is the impact of variation on learning?

**Self-directed**

- How do you experience autonomy over your own learning process?
- Do you have a concrete example of ‘self-directing’ your learning process?
- What is the impact of self-directing the learning process?

**Collaborative**

- How do you experience collaborative learning in the community of practice?
- Do you have a concrete example of collaborative learning?
- What is the impact of collaborating with students on learning?

**Future-oriented**

- How do you experience that education is future-oriented?
- Do you have a concrete example of this?
- What is the impact of future-oriented education on learning?
