# Supplementary material for: Preparing physiotherapists for the future: the development and evaluation of an innovative curriculum
Source: BMC Med Educ. 2025 Jan 17;25:83. doi: 10.1186/s12909-024-06537-1 (PMC11740659; doi:10.1186/s12909-024-06537-1)
Supplement: Supplementary file 2 — Supplementary Material 2. [file 12909_2024_6537_MOESM2_ESM.docx]

**Additional file 2: Interview guide teachers**

**Flexible**

- How do you experience flexibility in education, and do you have a concrete example of this?
- What is the impact of flexibility on the learning behavior of the students?
- How do you contribute to flexibility in your role as a teacher?

**Varied**

- How do you experience variety in education, and do you have a concrete example of this?
- What is the impact of variety on the learning behavior of the students?
- How do you contribute to variety in your role as a teacher?

**Self-directed**

- How do you experience students taking charge of their own learning process? Do you have a concrete example of this?
- What is the impact of self-directed learning on student behavior?
- How do you contribute to self-directed learning of students in your role as a teacher?

**Collaborative**

- How do you experience vertical learning between students, and do you have a concrete example of this?
- What is the impact of collaborative learning on student behavior?
- How do you contribute to collaborative learning in your role as a teacher?

**Future-oriented**

- How do you experience that education is future-oriented, and do you have a concrete example of this?
- What is the impact of future-oriented education on the learning behavior of the students?
- How do you contribute to future-oriented education in your role as a teacher?
