# Supplementary material for: Preparing physiotherapists for the future: the development and evaluation of an innovative curriculum
Source: BMC Med Educ. 2025 Jan 17;25:83. doi: 10.1186/s12909-024-06537-1 (PMC11740659; doi:10.1186/s12909-024-06537-1)
Supplement: Supplementary file 3 — Supplementary Material 3. [file 12909_2024_6537_MOESM3_ESM.docx]

| Questionnaire for teachers and students for the implementation of PACE | | | | | |
| --- | --- | --- | --- | --- | --- |
|  | Strongly disagree | |  | Strongly agree | |
|  | 1 | 2 | 3 | 4 | 5 |
| Varied & Flexible |  |  |  |  |  |
| 1. In education we work with varied clinical cases as they occur in professional practice. |  |  |  |  |  |
| 2. I can choose from a variety of clinical cases or learning activities and thus determine my own learning route. |  |  |  |  |  |
| 3. I can demonstrate the learning outcomes in my portfolio in various ways (product/video/performance etc). |  |  |  |  |  |
| 4. I can decide when I ask for feedback and in what form (written/conversation/combination). |  |  |  |  |  |
| 5. I can decide when my portfolio is ready for assessment. |  |  |  |  |  |
| 6. I experience sufficient variation in the learning activities such as lectures, workshops, written assignments, patient demonstrations. |  |  |  |  |  |
| 7. Learning coaches respond to my personal learning questions and meet my learning needs. |  |  |  |  |  |
| Self-directed & collaborative |  |  |  |  |  |
| 8. I am encouraged to take responsibility for my own learning process. |  |  |  |  |  |
| 9. I experience the added value of learning with students from different years (vertical CoP's). |  |  |  |  |  |
| 10. I receive useful feedback from teachers to improve my performance. |  |  |  |  |  |
| 11. I receive useful feedback from senior students to improve my performance. |  |  |  |  |  |
| 12. I receive useful feedback from peers (same study year) to improve my performance. |  |  |  |  |  |
| 13. I critically reflect on the feedback I have received and show what I have done with it. |  |  |  |  |  |
| 14. I feel safe in my learning group to share my learning experiences and demonstrate my learning results. |  |  |  |  |  |
| 15. My teachers have sufficient insight into my learning process. |  |  |  |  |  |
| 16. I feel connected to the students and teachers of my Community of Practice (CoP) |  |  |  |  |  |
| 17. I actively contribute to learning within my COP. |  |  |  |  |  |
| Future-oriented |  |  |  |  |  |
| 18. I get a clear picture of the future physiotherapist |  |  |  |  |  |
| 19. I am being prepared to adapt to the changing roles and tasks of the future physiotherapist. |  |  |  |  |  |
| 20. I develop learning skills for life-long learning. |  |  |  |  |  |
| 21. I am introduced to new technology for education and healthcare. |  |  |  |  |  |
| 22. Teachers approach me as a future colleague physiotherapist. |  |  |  |  |  |
